# Supplementary material for: Heterotypic Supramolecular Hydrogels Formed by Noncovalent Interactions in Inflammasomes
Source: Molecules. 2020 Dec 26;26(1):77. doi: 10.3390/molecules26010077 (PMC7795891; doi:10.3390/molecules26010077)
Supplement: Supplementary file 1 [file molecules-26-00077-s001.pdf]

Supporting Information

Heterotypic Supramolecular Hydrogels Formed by Noncovalent Interactions in Inflammasomes

Adrianna N. Shy, Huaimin Wang, Zhaoqianqi Feng and Bing Xu*

Department of Chemistry, Brandeis University, 415 South St. Waltham, MA 02453, USA
*Correspondence: bxu@brandeis.edu; Tel.: +01-781-736-5201

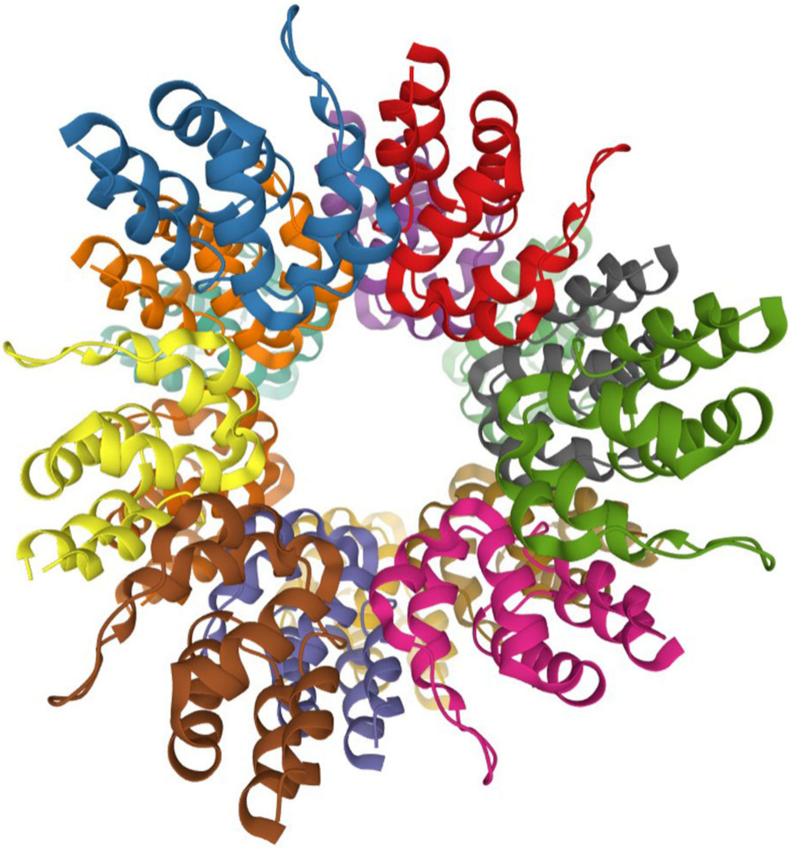

Figure S1. Structure of ASC-dependent inflammasome (PDB ID: 3J63)[40]. The sequence KKFKLKL is derived from amino acid numbers 21-27 and DALDLTD contains amino acid numbers 48-54.

General Synthesis Scheme of Nap-FFKKFKLKL

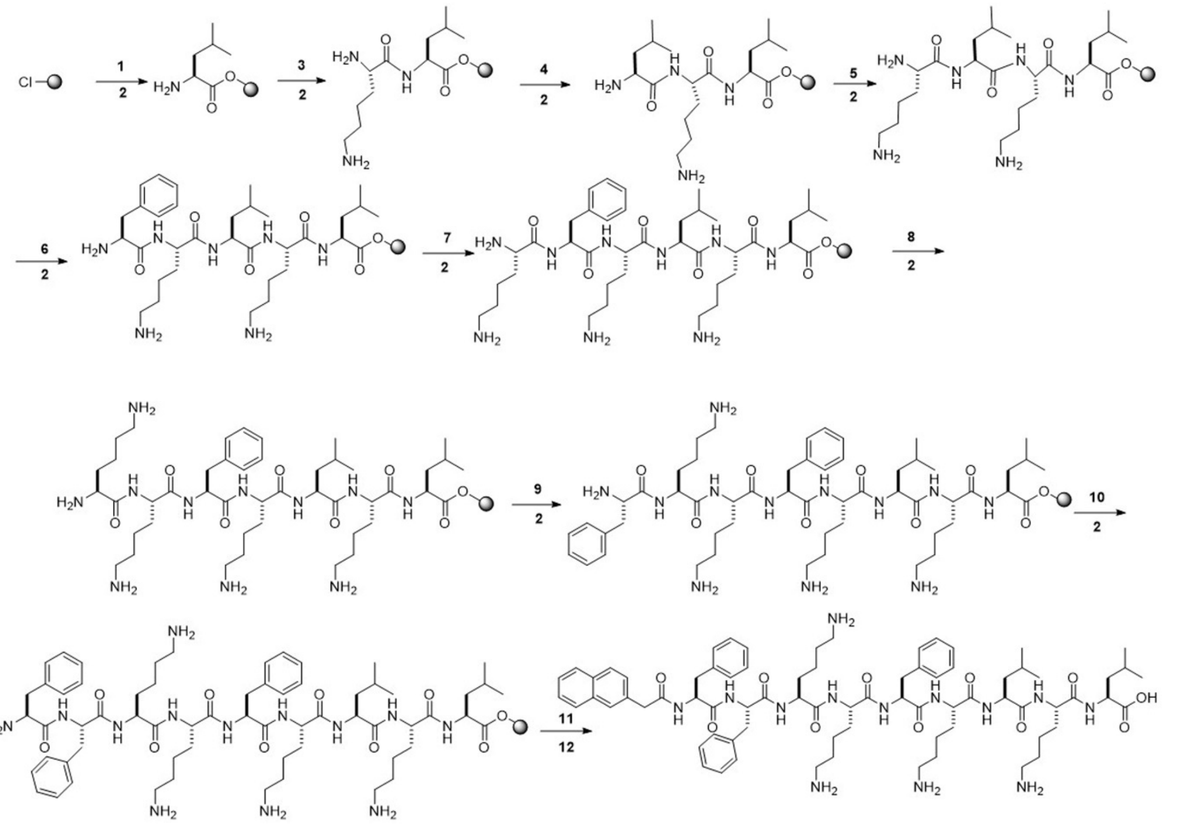

Scheme S1. (1) addition of leucine and DIEA. (2) addition of 20% piperidine. (3) addition of lysine, DIEA and HBTU. (4) addition of leucine, DIEA, and HBTU. (5) addition of lysine, DIEA, and HBTU. (6) addition of phenylalanine, DIEA and HBTU. (7) addition of lysine, DIEA, and HBTU. (8) addition of lysine, DIEA, and HBTU. (9) addition of phenylalanine, DIEA and HBTU. (10) addition of phenylalanine, DIEA and HBTU. (11) addition of 2-naphthalene acetic acid, DIEA and HBTU. (12) cleavage of Nap-FFKKFKLKL with 99% TFA. (All amino acids used are Fmoc protected and side chain protected where necessary.

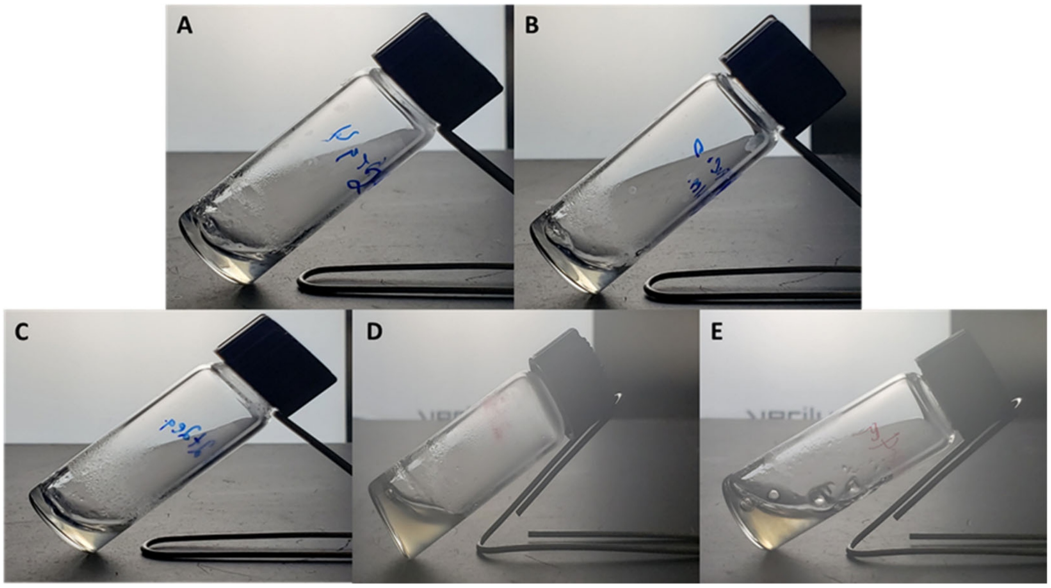

Figure S2. **2** (0.4 wt%) with 0.5, 1, 2, 3, and 4 molar equivalents of **4** in water after 24 hrs. All are viscous solutions containing precipitates.

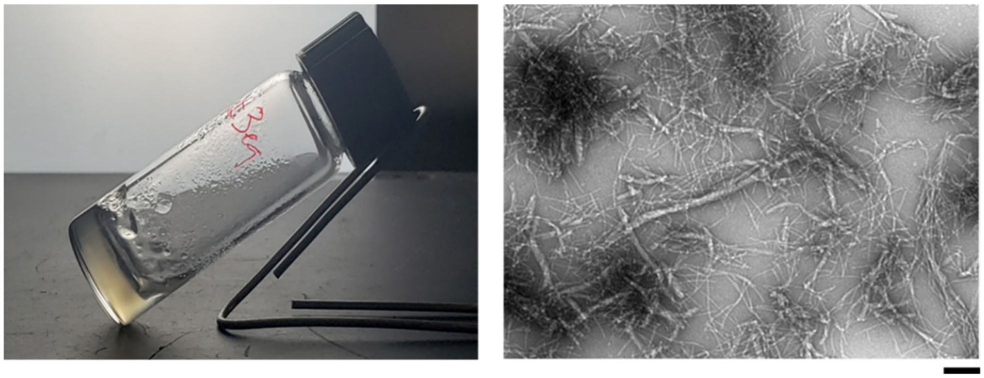

Figure S3. Optical and TEM images of **2** (0.4 wt%) + 3 equiv. of **4** after 27 days (mixed in water). Scale bar is 100nm.

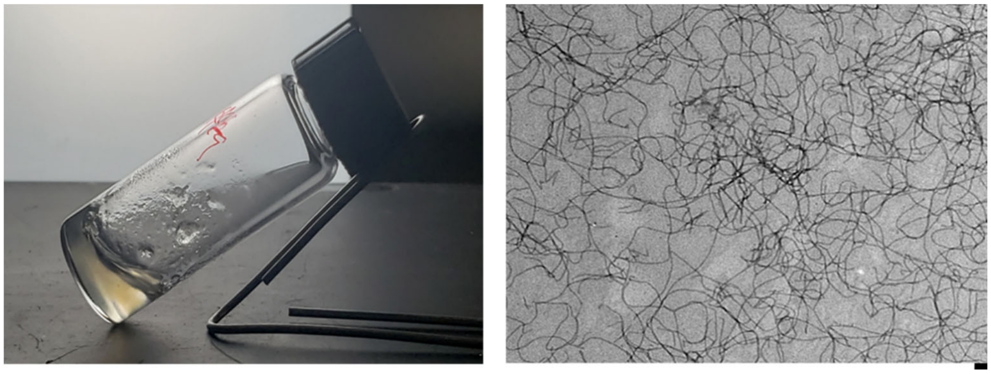

Figure S4. Optical and TEM images of **2** (0.4 wt%) + **4** equiv. of **4** after 27 days (mixed in water). Scale bar is 100nm.

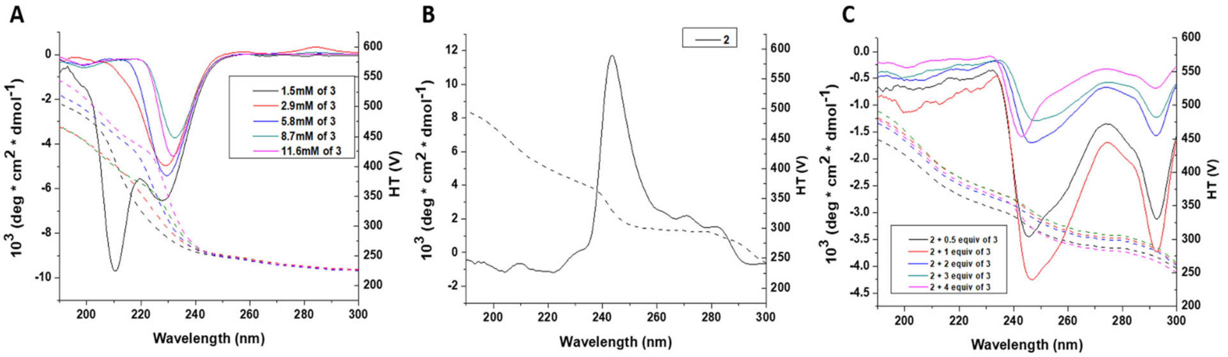

Figure S5. Circular dichroism of compounds **2** and **3** with high tension. Dotted lines correspond to the same colors as the solid lines in the legend.

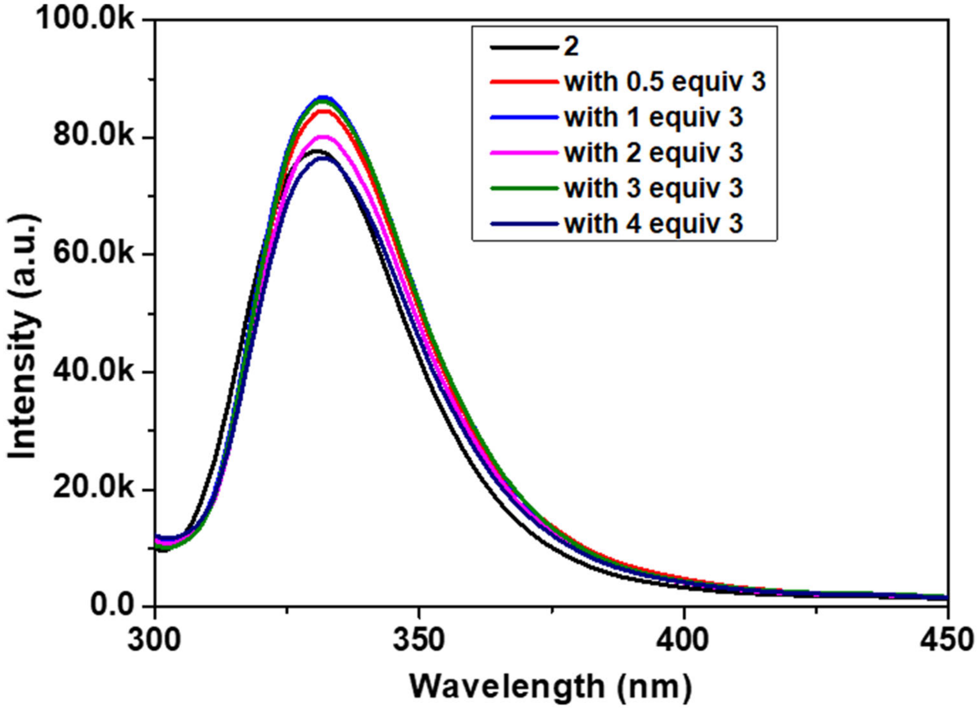

Figure S6. Fluorescence of NapFFKKFKLKL (**2**) without and with 0.5, 1, 2, 3 and 4 equivalents of AcDALDLTD (**3**).
